# 

**Published:** 1893-12

**Authors:** 


### CONTENTS.

CLINICAL LECTURE—
Case of Cleft Palate, Sarcomatous Testis, Double Genu-Valgus. By Thomas H. Manley, M.D., New York.......... 577
ORIGINAL COMMUNICATIONS—
Operative Procedures for Carcinomatous Tumors of the Breast. By J. McFadden Gaston, M.D., Atlanta, Ga.......... 579
Chancroids of the Female Generative Organs. By E. C. , A.B., M.D., Atlanta, Ga.......... 587
The Best Treatment for the Umbilical Cord. By C. D. Hurt, M.D., Atlanta, Ga. 594
SOCIETY REPORTS—
Southern Surgical and Gynecological Association.......... 595
CORRESPONDENCE—
New York Letter.......... 609
EDITORIAL—
Report of the Third Hyderabad Chloroform Commission.......... 615
Southern Surgical and Gynecological Association.......... 617
The Legislature and the Bill.......... 619
SELECTIONS AND ABSTRACTS—
General Medicine and Therapeutics.......... 621
Skin Diseases and Syphilis.......... 627
Surgery.......... 631
Practical Chemistry and Pharmacy.......... 636
MEDICAL ITEMS.......... 637
BOOK REVIEWS.......... 639
ADVERTISERS' INDEX TO ADVERTISEMENTS.......... xii
CLASSIFIED INDEX TO ADVERTISEMENTS.......... xxxix
ITEMS OF INTEREST.......... x, xi, xii
PREMIUM OFFERS.......... xxxviii

